# Correlation between task-based checklists and global rating scores in undergraduate objective structured clinical examinations in Saudi Arabia: a 1-year comparative study

**DOI:** 10.3352/jeehp.2025.22.19

**Published:** 2025-06-19

**Authors:** Uzma Khan, Yasir Naseem Khan

**Affiliations:** 1 Department of Clinical Sciences, College of Medicine, Al Rayan National Colleges, Medina Al-Munawara, Saudi Arabia; 2 Department of Basic Sciences, College of Medicine, Al Rayan National Colleges, Medina Al-Munawara, Saudi Arabia; The Catholic University of Korea, Korea

**Keywords:** Checklist, Clinical competence, General practice, Medical students, Saudi Arabia

## Abstract

**Purpose:**

This study investigated the correlation between task-based checklist scores and global rating scores (GRS) in objective structured clinical examinations (OSCEs) for fourth-year undergraduate medical students and aimed to determine whether both methods can be reliably used in a standard setting.

**Methods:**

A comparative observational study was conducted at Al Rayan College of Medicine, Saudi Arabia, involving 93 fourth-year students during the 2023–2024 academic year. OSCEs from 2 General Practice courses were analyzed, each comprising 10 stations assessing clinical competencies. Students were scored using both task-specific checklists and holistic 5-point GRS. Reliability was evaluated using Cronbach’s α, and the relationship between the 2 scoring methods was assessed using the coefficient of determination (R^2^). Ethical approval and informed consent were obtained.

**Results:**

The mean OSCE score was 76.7 in Course 1 (Cronbach’s α=0.85) and 73.0 in Course 2 (Cronbach’s α=0.81). R^2^ values varied by station and competency. Strong correlations were observed in procedural and management skills (R^2^ up to 0.87), while weaker correlations appeared in history-taking stations (R^2^ as low as 0.35). The variability across stations highlighted the context-dependence of alignment between checklist and GRS methods.

**Conclusion:**

Both checklists and GRS exhibit reliable psychometric properties. Their combined use improves validity in OSCE scoring, but station-specific application is recommended. Checklists may anchor pass/fail decisions, while GRS may assist in assessing borderline performance. This hybrid model increases fairness and reflects clinical authenticity in competency-based assessment.

## Graphical abstract

[Fig f1-jeehp-22-19]

## Introduction

### Background/rationale

Since its introduction by Harden and Gleeson [1] in 1979, the objective structured clinical examination (OSCE) has become a widely accepted tool for assessing clinical competencies. The control over classic variables in the patients and examiners and the clear definitions of the skills and attributes being evaluated are the two main factors contributing to its success. Its objectivity, consistency, ability to evaluate a variety of clinical scenarios practically, and its capacity to evaluate a wide range of skills in short time frame have made OSCEs a valuable approach in medical education [2].

Objectivity and reliability are added by incorporating task-based checklists into OSCEs [2]. These checklists are prepared by a panel of experts who agree on the selection of critical items considered essential for examinees to perform in a specific manner. However, this combination of items often fails to capture the reason for poor performance, such as gaps in knowledge, skill, or reasoning [3].

Global rating scores (GRS) are an important element of OSCE measurement and exhibit good psychometric properties. They give holistic judgments of performance, not confined to specific behaviors or actions that must be performed, and are based on 3 levels of cognitive skill development: novice, acceptable performance, and expert [4]. GRS are susceptible to subjectivity and bias and are not reliable because the checklist performance may influence the examiner’s perception of the examinee’s overall competence, rather than being a true reflection of their skills. Despite widespread use of both methods, debate continues about their validity and reliability.

The metric used to analyze the reliability of OSCE is Cronbach’s α, which reflects the internal consistency of the test. The overall value for alpha that is usually regarded as acceptable in this type of assessment is 0.7 or above [4]. The coefficient of determination (R^2^) indicates the correlation between the expert-judged global ratings and the expert checklist scores. An R^2^ value >0.5 suggests a reasonably good relationship between checklist scores and global grades. More specifically, R^2^ <0.3 indicates a weak correlation, 0.5 to 0.6 is considered moderate, and R^2^ >0.7 suggests a strong correlation [5].

Since an OSCE is a standardized simulation of real clinical practice, it effectively measures the essential knowledge, skills, and attitudes required for competent medical practice. However, there is considerable debate in the literature regarding the validity of checklists versus domain-based global scoring, but few studies have directly compared these 2 methods to determine whether checklist scoring can be replaced with domain-based global ratings or whether combining the 2 methodologies will be helpful.

To address this knowledge gap, and to ensure the validity of OSCE exams at the College of Medicine, Al Rayan National Colleges, we investigated our own undergraduate Year 4 OSCE. By comparing task-based checklist scores with GRS across 20 stations assessing various competencies, we aimed to determine their correlation and identify any areas where one method may outperform the other. Our findings aim to convey efforts to improve OSCE design and standard-setting practices.

### Objectives

The purpose of this study was to determine the correlations between the standard task-based checklist and GRS as scoring methods in the OSCE for General Practice courses in fourth-year medical students over 1 year.

## Methods

### Ethics statement

Approval was obtained from the Al Rayan College Research Ethics Committee (REC) for the collection and publication of student data (approval no., HA-03-M-122-046). Informed consent was received from students, and permission from the dean was granted to use students’ results for research and quality improvement, ensuring all personal identifiers were removed.

### Study design

This was an observational comparative study, conducted at the Department of Clinical Science, Al Rayan College of Medicine, involving year 4 students over 2 consecutive courses in their fourth year of medical studies. Physician-marked checklist scores and holistic GRS were compared. The results from 20 stations, 10 from each course, were analyzed. IBM SPSS Statistics for Windows ver. 20.0 (IBM Corp.) was used for analysis. OSCE reliability was first checked using Cronbach’s α, and then the correlation coefficient of determination was used to identify correlations between the scoring methods.

### Setting

The study was conducted at the Department of Clinical Science, Al Rayan College of Medicine, located Madina Al Munawara, Saudi Arabia. It focused on Year 4 undergraduate medical students enrolled in the General Practice 1 and 2 courses during the academic year 2023–2024. Data was collected from the final OSCEs administered at the end of each course. The OSCE was conducted in a state-of-the-art simulation lab and consisted of 10 main stations with an active rest station. Examinees had 6 minutes to complete each station. Performance was scored using 10 predefined competencies aligned with course learning outcomes, designed under the competence specifications for Saudi medical graduates (Saudi Meds). The following skills were assessed: (1) history taking, (2) physical examination, (3) analysis and interpretation of findings, (4) communication, (5) suggestion of appropriate investigations, (6) listing relevant differential diagnoses, and (7) patient management plan development. The values assessment included 3 competencies: (1) ethical rules and confidentiality, (2) taking and maintaining consent, and (3) time management. These competencies were assessed at each station [6]. During the active stations, examiners used 2 types of scoring systems, a task-specific checklist during the performance and GRS at the end (Supplement 1). The checklist included detailed steps for each station, which the candidate needed to complete (Supplement 2).

Three panels of experts were established, each having 10 stations and 2 circuits of students. Students rotated through the stations, completing a single circuit in an anticlockwise manner. Each student was examined by a single examiner at each station except for the radiological interpretation station, which was monitored by a silent invigilator and students recorded their answers on an answer sheet. The standardized patients were briefed verbally, with written information provided for their respective stations. Examiners received guidance on awarding global scores, and all examiners marking the same stations participated in an online orientation session conducted by the course coordinator the day before the OSCE.

### Participants

This study included a total of 93 year 4 students (27 male and 66 female) undertaking the final OSCEs in the general practice 1 & 2 course. All students who passed through the General Practice courses were included in the study.

### Variables

Checklist scores and GRS were outcome variables.

### Data sources/measurement

Checklist scores were obtained from task-based checklists developed by expert panels for each OSCE station and consisted of critical items that students were required to perform correctly during OSCE. The total score for each checklist was calculated by summing the scores of all items of the checklist. The checklists were content-validated by subject experts, and reliability was assessed using Cronbach’s α, with values of 0.7 considered acceptable.

GRS were assigned by trained examiners using a holistic scoring rubric for each station using a 5-point scale of unsatisfactory=1, borderline (fail)=2, pass=3, good=4, and excellent=5. Examiners were guided about the rubric at the start of OSCE. Global ratings were validated by inter-rater reliability testing.

### Bias

Potential biases include variations in examiners and patient interactions. These were controlled by giving an orientation briefing before the start of the OSCE by explaining the examiners “assessment criteria and scoring checklists” and giving them opportunities for clarification. Standardized patients were trained according to scripted scenarios.

### Study size

All students were eligible to take the exams; no student withdrew therefore all students (n=93) were included in the study. As a complete census of the eligible student cohort was analyzed, sampling bias was not a concern. A post hoc power analysis using the observed correlation of ρ^2^=0.3532 (effect size=0.59, 91 examinees) indicated >0.99 power (α=0.05) using G*Power (Heinrich-Heine-Universität Düsseldorf). When ρ^2^ was set to 0.85, the effect size to 0.59, and the number of examinees to 91, the power was more than 0.99 (α=0.05).

### Statistical methods

Descriptive statistics of the 2 end course results were analyzed, including the man, standard deviation (SD), Cronbach’s α for station reliability, R^2^, Inter-grade discrimination, and between-group variation. The relationship between examiner checklist ratings and global domain-based ratings was assessed using R^2^, which was calculated within SPSS using the output from the exam. Spearman’s rank correlation was initially considered due to the ordinal nature of the rating scales. The reported correlation values in subsequent tables refer to coefficient of determination (R^2^) unless specified, as it is commonly used in the literature [7].

## Results

### Participants

All 93 fourth-year students agreed to participate after informed consent was obtained. No student declined to participate or withdrew from the study. Among these, 27 were male (29%) and 66 were female (71%).

### Main results

The General Practice 1 OSCE result showed that average achieved score was 76.7 with an SD of 4.54. The minimum total score achieved was 10.00, and the highest was 21.57, out of a total score of 24 (89.8%). The percentage of students failing the OSCE was 3.22 and the variance in the score was 20.58. Cronbach’s α for inter-station reliability in General Practice 1 OSCE was 0.85, indicating good internal consistency among the stations (Dataset 1).

At the end of the first half of the year, the first OSCE results are shown in Table 1 with details of R^2^ values between physician-marked checklists and GRS across 10 stations. The R^2^ values ranged from 0.35 for examination stations, indicating a weak correlation, to 0.85 for station 3, one of the history taking stations, demonstrating a strong correlation.

The average score for the second OSCE at the completion of the General Practice 2 course was 73, indicating that students performed moderately across the stations. The SD was 2.48, suggesting relatively low variability in scores among students. The median score was 17.60 (73%). The minimum mark achieved was 11.89 (49.5%), while the maximum was 22.38 (93%). Cronbach’s α was calculated as 0.81, indicating good internal consistency among the OSCE stations and suggesting that the stations reliably measured similar competencies. No significant differences in performance between different groups or cohorts within this OSCE were noted, as is evident by between-group variation of 0.00. A detailed analysis of all the metrics of the OSCE is shown in Table 2 and Dataset 2.

In General Practice 2, Stations 1 and 8 (both history taking) showed the lowest correlation (R^2^ values of 0.40 and 0.45), indicating weak agreement between checklist scores and global ratings for these stations. Conversely, Stations 3 and 6 (management stations) exhibited the highest correlation (R^2^=0.74 and 0.86 respectively), indicating strong agreement between checklist scores and global ratings.

The coefficients of determination for stations of the OSCE in General Practice courses in terms of competencies are shown below in Table 3.

Table 3 gives R^2^ values for OSCE stations grouped by competencies across the two courses. In General Practice 1, history-taking skills demonstrated a moderate to strong correlation between checklist scores and global ratings (mean R^2^=0.66, SD=0.20), while procedural skills showed the highest correlation (mean R^2^=0.68, SD=0.14). In contrast, physical examination skills exhibited stronger correlations in General Practice 2 (mean R^2^=0.62, SD=0.05), alongside management/prescribing skills, which demonstrated very high alignment (mean R^2^=0.80, SD=0.09).

## Discussion

### Key results

The study found strong and positive correlations between physician checklists and domain-based scores. Specifically, the Cronbach’s α values of 0.85 and 0.81 indicated acceptable internal consistency for both General Practice 1 and General Practice 2 OSCEs, respectively. Furthermore, the mean R^2^ showed a positive relationship between the 2 scoring methods across most stations, although the strength of the correlation varied by the station and the course.

### Interpretation

While checklists offer detailed task-specific evaluations, and global ratings provide holistic judgement of overall competency, both in fact can validly and reliably assess students’ performance. This agrees with previous studies using simulation-based OSCEs proving that both GRS and checklists can effectively evaluate performance [8]. This finding is further supported by the study of Zoller et al. [9] in 2021, which demonstrated that GRS represent a promising tool to objectively assess technical skills in simulation training, with high construct validity and interrater reliability.

Our study identified significant variability across competencies and between the 2 courses particularly in history taking stations and procedural skills assessment, a finding that does not align with a study by Sim et al. [2] in 2015 at the University of Malaysia, who found statistically significant correlations between all station types including procedural skills. This suggests that the relationship between checklist scores and global ratings may be more complex and context-dependent than previously understood, warranting further investigation into the factors contributing to these discrepancies. Variations in history-taking results could be due to differences in examiners and/or standardized patients as the OSCE runs across 3 circuits at the same time; the examiners and standardized patients were different for each circuit. However, the examiners and standardized patients were the same throughout the rounds.

The use of global ratings on a 5-point scale with anchors (unsatisfactory, borderline, pass, good, excellent) could possibly cause Station 6 to be the lowest. For the procedural skill station, a more detailed assessment is required, and a finer rating scale is needed to discriminate among candidates with different levels of competency. A detailed checklist can cater to this need, but it is important to remember that global ratings allow examiners to judge whether students’ performance reflects a natural encounter between a physician and a patient in a practical setting in terms of a student’s level of confidence and how much trust he or she has gained from the patient. The lower correlation observed in General Practice 2 indicates that procedural skills may benefit from integrating both scoring methods to capture nuances in competency levels.

### Comparison with previous studies

A comparison with a recent study by Mahmoud [7] in 2023 underscores the differences in how competencies were assessed across courses and points toward the potential need for refining station designs. Checklists were previously thought to measure information-gathering and completeness more effectively, while domain-based ratings are more suited to the appraisal of communication skills, empathy, consultation structure, and general competence [7]. This distinction underscores the need to employ both assessment methods to capture the full spectrum of competencies.

The observed improvement in correlation in management competencies towards the end of the course in 2nd OSCE is explained by the fact that students gain experience with time. This finding is supported by Turner et al. [10], who explained that as the students become more experienced and confident, they are likely to combine acquired knowledge, technical, communication, and holistic organizational skills.

After studying results from several studies, Hodges et al. [11] concluded that checklists do not measure the increasing level of experience and reliance on global ratings is mandatory in OSCE. Specifically, our results for data interpretation skills align with the observations of Abass and Ahmed [12] who demonstrated that the correlation between checklist scores and GRS scores became stronger as students gained experience. We observed that mean R^2^ values increased for data interpretation stations from 0.54 in the first OSCE to 0.66 in the second course [12]. This suggests that, as students progress and gain experience in data interpretation, the checklist and GRS become more aligned in their assessment of competence.

These findings have important implications for OSCE standard-setting. The strong correlation between the two methods for most stations validates the use of both systems. However, in some stations with weaker agreement, such as history taking, the study data suggest that a checklist should anchor pass/fail decisions, while GRS could help make decisions about borderline cases.

### Limitations

Limitations of the study include variability among examiners and standardized patients, which can be a source of bias in scoring, especially for the history-taking and examination stations. Moreover, the single-institution design of this study limits its generalizability. Despite these constraints, the consistent correlation across most courses over the span of 1 year supports the validity of both scoring methods.

### Generalizability

The generalizability of the results may be limited to similar educational settings using a structured OSCE with competency-based frameworks. While the correlation was robust across most stations, educators in other health sectors like nursing, pharmacy and allied health should validate these results in their contexts.

### Conclusion

The study results support using both checklists and GRS in OSCE standard-setting, with station-specific weighting based on their correlation strength. For stations with strong correlations, standards can integrate both methods seamlessly. For weaker-correlation stations, checklists should anchor decisions, with GRS resolving borderline cases. This approach balances reproducibility with clinical realism, advancing fairness in competency assessment.

## Figures and Tables

**Figure f1-jeehp-22-19:**
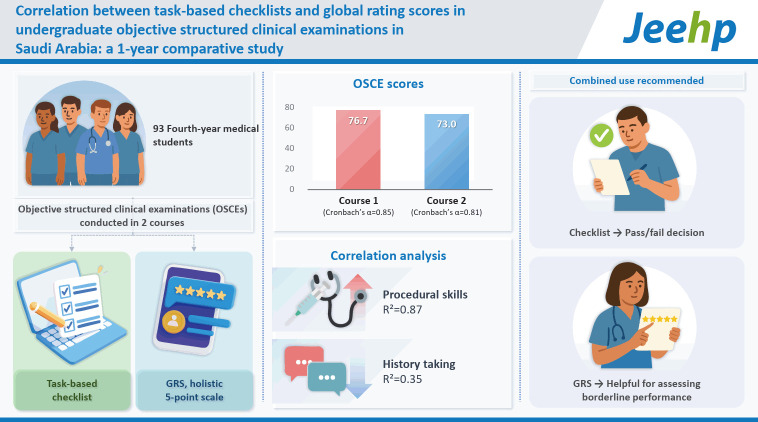


**Table 1.** Distribution of results for the OSCE for the first course

Station	Average (%)	Standard deviation	Cronbach’s α if item deleted	Coefficient of determination R^2^	Inter-grade discrimination	Between-group variation (%)
Station 1: History	70	0.23	0.76	0.44	2.16	0.00
Station 2: History	81	0.29	0.71	0.67	4.32	0.04
Station 3: History	35	0.52	0.87	0.85	5.52	0.01
Station 4: Examination	98	0.15	0.81	0.35	3.01	0.00
Station 5: Examination	88	0.31	0.79	0.62	4.67	0.04
Station 6: Data interpretation	79	0.24	0.82	0.51	3.89	0.01
Station 7: Data interpretation	77	1.13	0.86	0.57	3.22	0.00
Station 8: Management	71	0.34	0.89	0.52	2.01	0.01
Station 9: Procedure	89	0.37	0.70	0.79	5.08	0.01
Station 10: Procedure	78	0.29	0.89	0.59	3.12	0.00

OSCE, objective structured clinical examination.

**Table 2.** Detailed results for the OSCE for the second course

Station	Average (%)	Standard deviation	Cronbach α if item deleted	Coefficient of determination R^2^	Inter-grade discrimination	Between-group variation (%)
Station 1: History	74	0.33	0.83	0.40	2.95	0.01
Station 2: Examination	87	0.29	0.83	0.65	3.42	0.00
Station 3: Management	76	0.39	0.84	0.74	3.21	0.01
Station 4: Procedure	78	0.44	0.84	0.50	3.63	0.01
Station 5: Procedure	85	0.31	0.82	0.57	3.18	0.01
Station 6: Management	65	0.56	0.87	0.87	4.68	0.01
Station 7: Consultation	78	0.10	0.83	0.69	2.96	0.02
Station 8: History	65	0.36	0.83	0.45	3.30	0.00
Station 9: Examination	85	0.24	0.82	0.58	2.25	0.01
Station 10: Data interpretation	42	0.62	0.88	0.66	5.40	0.02

OSCE, objective structured clinical examination.

**Table 3.** Coefficient of determination (R²) for OSCE stations in General Practice courses by competencies

Competencies	1st OSCE (N=93)		2nd OSCE (N=93)	
	No. of stations	Mean R^2^ values±SD	No. of stations	Mean R^2^ values±SD
History skills	3	0.66±0.20	2	0.43±0.03
Physical examination skills	2	0.48±0.18	2	0.62±0.05
Procedural skills	2	0.68±0.14	2	0.53±0.04
Data interpretation skills	2	0.54±0.03	1	0.66
Management/prescribing skills	1	0.51	2	0.80±0.09
Consultation			1	0.69

OSCE, objective structured clinical examination; SD, standard deviation.

## Footnotes

**Data availability**
Data files are available from https://doi.org/10.7910/DVN/8E1NBY The dataset 1 cited within the main text is Table 1, similarly dataset 2 cited as Table 2. Dataset 1. General Practice 1 OSCE results.

Dataset 2. General Practice 2 OSCE results.

## Supplementary materials

Supplement 1. Assessment unit OSCE exam: year semester date station.

Supplement 2. Assessment unit OSCE exam: year 4 semester 2 date station.

Supplement 3. Audio recording of the abstract.
