# Supplementary material for: Correlation between task-based checklists and global rating scores in undergraduate objective structured clinical examinations in Saudi Arabia: a 1-year comparative study
Source: J Educ Eval Health Prof. 2025 Jun 19;22:19. doi: 10.3352/jeehp.2025.22.19 (PMC12365684; doi:10.3352/jeehp.2025.22.19)
Supplement: Supplementary file 3 — Supplement 1. Assessment unit OSCE exam: year semester date station. [file jeehp-22-19-suppl1.pdf]

ASSESSMENT UNIT OSCE EXAM.
YEAR 4 SEMESTER 2 DATE STATION (--)

Marking Form

***Assessors should circle the appropriate score box for each* item of the checklist.**

**Candidate's name:**....................................	**ID number:**..				
**Checklist items**	**Marks**				
**Q1. Introduction and opening the session**					
Introduction, orientation and ensures that patient is comfortable.	**0**	**1**	**2**	**3**	
Establishes name, age, and occupation.	**0**	**1**	**2**	**3**	
**Q2. Analysis of the complaint: Ask about**					
Onset, course, and duration of the weakness	**0**	**1**	**2**	**3**	
Is the weakness only in the lower limbs (or there is weakness in other part of the body)	**0**	**2**			
Is the weakness show any diurnal variation?	**0**	**1**			
Is weakness associated with sensory symptoms?	**0**	**2**			
Is the weakness associated with sphincter disturbances?	**0**	**2**			
Is the weakness associated with back pain?	**0**	**1**			
Is the weakness associated other body system affection	**0**	**1**			
**Q3. Past history Each item =1 mark**					
Similar condition,<br>Medical / Surgical disease,<br>Blood transfusions,<br>Drug history.	**0**	**1**	**2**	**3**	**4**
**Q4. Family history Each item =1 mark**					
Consanguinity,	**0**	**1**	**2**	**3**	

Similar disease,<br><br>Other chronic conditions					
**Q5. Social history Each item =1 mark**					
Special habit (smoking, alcohol, elicited drugs)<br><br>Traveling abroad	**0**	**1**	**2**		
**Q6.** Anatomically where is the lesion? And why? Justify your answer. 2marks for each question					
in the spinal cord.<br><br>As affection only in both legs, with sensory and sphincter lesion	**0**	**2**	**4**		
**Q7. Can you list at least FOUR causes that might result in a lesion at this location?<br><br>Any four each carries 1 mark.**					
Trauma, tumour, infection, disc prolapse, infarction, haemorrhage, syringomyelia, vertebral fracture.	**0**	**1**	**2**	**3**	**4**
**Q8. What are the characteristics of spinal cord lesions? 4 points are required. each point=1mark**					
It is upper motor neurons lesion characterized by.<br><br>Hyper Tonia<br><br>Hyperreflexia<br><br>Late muscle wasting<br><br>Positive planter Flexion sign (Babinski)	**0**	**1**	**2**	**3**	**4**
**Total Marks**	**/ 38**				

**Candidate Performance Global rating**				
1	2	3	4	5
**Unsatisfactory**	**Borderline (fail)**	**Pass**	**Good**	**Excellent**

**Assessor Name: -----------------------------**	**Signature: ----------------------------------**
