# Supplementary material for: Correlation between task-based checklists and global rating scores in undergraduate objective structured clinical examinations in Saudi Arabia: a 1-year comparative study
Source: J Educ Eval Health Prof. 2025 Jun 19;22:19. doi: 10.3352/jeehp.2025.22.19 (PMC12365684; doi:10.3352/jeehp.2025.22.19)
Supplement: Supplementary file 4 — Supplement 2. Assessment unit OSCE exam: year 4 semester 2 date station. [file jeehp-22-19-suppl2.pdf]

ASSESSMENT UNIT.OSCE EXAM.
YEAR SEMSTER DATE STATION (--)

ORGANIZER FORM

STATION DISCIPLINE:
STATION SUBDOMAIN:
STATION DESIGNER /DOMAIN COORDINATOR: ----------------
STATION TYPE:
(Clinical Task/ SP/Visual Recognition/ Written or Oral Task/ Linked Procedure)
STATION COMPTENCIES/ILOS :

Example:

- To assess the candidate`s skills to apply------------
- To assess the candidate's skills to perform ---------

Assessor: **One** Assessor must attend

Simulator: **None**/**One** simulator must attend

Time: **6** minutes

Resources and Requirements needed for setting up of the station:

(Patients/Simulated Patients, Beds, Room set-up, Equipment etc---)

- **Two/Three** chairs.
- **One** Desk
- **Instruction and marking forms:**

o **Candidate's instruction sheet if needed** (Rest station version) (**None/One** copy)

o **Candidate's instructions sheet** on desk (**One** copy)

o **Simulator Scenario sheet** (**None /One** copy)

o **Assessor instructions** sheet (**One** copy)

o **Marking sheets one hard copy will be provided for assessor and students** (google forms) (hard copies will be provided as spare if needed)

o **Requirements/Equipment** if needed (please state):
1-
2-

ASSESSOR FORM

Time: 6 minutes

Assessor Instructions:

Assessors are requested to attend 20 minutes before the exam starts to revise their stations and counsel any comments with the domain coordinator.

Step 1:

- Kindly check the station instructions, and marking forms.
- Make sure that all required supplements for the station are available.

Step 2:

- Check the candidate's ID, Write the candidate's name and number on the marking sheet.

Step 3:

- Ask the candidate to read the instruction sheet and perform the requested actions accordingly.
- No facial expressions or performance remarks are allowed.
- Do not give the candidate any comment or clue to an answer.
- Candidate is not allowed to direct questions to you.
- Remind candidates that time is passing if they do not commence the test after two minutes.) may vary according to station form
- (If candidates do not begin their report by the last-minute prompt them by saying 'And what have you found'.) may vary according to station form
- Add questions here: Q1/Q2etc,,,

Step 4:

- Use the provided **Mark Sheet** to rate the candidate's performance.
- Rate the overall candidate performance using the provided scale.
- ***Checklist Rubric***
  - When only two points scale; it means either fully done or not done to fulfill minimum required
  - When multiple responses/ actions are needed give one mark for each response/ action
  - When a scale is used for marking certain skill: use the following descriptor for marking:

0	*Not done or* UNACCEPTABLE
1	BORDERLINE UNACCEPTABLE
2	BORDERLINE ACCEPTABLE
3	ACCEPTABLE
4	ABOVE the level expected

- **Feedback** notes are needed on candidates' performance. kindly provide in the supplied form. These notes might be discussed with candidate's post-exam especially underachievers.

ASSESSMENT UNIT.OSCE EXAM.
YEAR SEMSTER DATE STATION (--)

CANDIDATE FORM 1

Time: 6 minutes

Candidate Instructions

Scenario/Question

Your task at this station is to

Q1

Q2

Candidate Form 2

Supplementary Material (IF NEEDED)

Marking Form

ASSESSMENT UNIT.OSCE EXAM.
YEAR SEMSTER DATE STATION (--)

Assessors should circle the appropriate score box for each item of the checklist.

Candidate's name:...................................			ID number:...................................….......					
**Checklist items**				**Marks**				
**Q1.**								
				0	1	2	3	4
**Q2.**								
				0	1	2	3	4
**Q3.**								
				0	1	2	3	4
**Q4.**								
				0	1	2	3	4
**Q5.**								
				0	1	2	3	4
**Q6.**								
				0	1	2	3	4
**Total Marks**				/				
**Candidate Performance Global rating**								
1	2	3	4	5				
**Unsatisfactory**	**Borderline (fail)**	**Pass**	**Good**	**Excellent**				
**Assessor Name:** ------------------------------			**Signature:**------------------------------------					

SIMULATOR(STANDARDIZED PATIENT) FORM

ROLE

SCENARIO

ASSESSMENT UNIT.OSCE EXAM.
YEAR SEMSTER DATE STATION (--)

ASSESSOR FEEDBACK ON CANDIDATE PERFORMANCE FORM

No	ITEM	Yes	No
1.	**TIME: HE COULD PERFORM TASKS ON TIME**		
2.	**ATTITUDE: APPROPRIATE**		
3.	**TASK: HIS TASK PERFORMANCE WAS APPROPRIATE TO THE COMPETENCY ASSESSED**		

Candidates performed poorly in the following areas:

Recommendations for Improvement (If any)

Assessor name: Assessor Signature:

ASSESSMENT UNIT.OSCE EXAM.
YEAR SEMSTER DATE STATION (--)

ASSESSOR FEEDBACK ON STATION FORM

TIME --

SETTING --

CLARITY --

TASK DIFFICULTY ---

APPROPRIATENESS OF TASKS FOR COMPETENCY ASSESSMENT AND COMPLETION--
----

APPROPRIATE PRE-EXAM ORIENTATION AND ORGANIZATION---

OTHERS--

Assessor name: Assessor Signature:

STATION STANDARDIZATION FORM (DESIGNER/ COORDINATOR)

STATION(1)/ MINIMAL ACCEPTABLE PASSING SCORE	RATER 1 (STATION DESIGNER)	RATER 2 (STATION REVIEWER)	AVERAGE SCORE
QUESTION 1			
RATIONALE			
QUESTION 2			
RATIONALE			
QUESTION 3			
RATIONALE			
QUESTION 4			
RATIONALE			
QUESTION 5			
RATIONALE			
QUESTION 6			
RATIONALE			
TOTAL SCORE			

MINIMAL ACCEPTABLE PASSING SCORE

REFERS TO THE LOWEST POSSIBLE SCORE ON A STANDARDIZED STATION ASSESSMENT THAT A BORDERLINE STUDENT MUST EARN TO "PASS".
